# Association Between COVID-19 Relief Funds and Hospital Characteristics in the US

**DOI:** 10.1001/jamahealthforum.2021.3325

**Published:** 2021-10-22

**Authors:** Jonathan Cantor, Nabeel Qureshi, Brian Briscombe, Justin Chapman, Christopher M. Whaley

**Affiliations:** 1 RAND Corporation, Santa Monica, California; 2 RAND Corporation, Arlington, Virginia

## Abstract

**Importance:**

In response to financial stress created by the reduction in care during the COVID-19 pandemic, hospitals received financial assistance through the Coronavirus Aid, Relief, and Economic Security (CARES) Act program. To date, the allocation of CARES Act funding is not well understood.

**Objective:**

To examine the disbursement of the High-Impact Distribution CARES Act funds and the association between financial assistance and hospital-level financial resources prior to the COVID-19 pandemic.

**Design, Setting, and Participants:**

This cross-sectional analysis of US-based hospitals and health systems assesses the hospital characteristics associated with CARES Act funding with linear regression models using linked hospital and health system–level information on CARES Act funding with hospital characteristics from Hospital Cost Report data.

**Exposures:**

Hospital and health system CARES Act financial assistance.

**Main Outcomes and Measures:**

Hospital and health system affiliation, status, and financial health prior to the COVID-19 pandemic. Data analysis took place from December 2020 through June 2021.

**Results:**

The analysis included 952 hospital-level entities with an average payment of $33.6 million, most of which was received during the first payment round. Wide ranges existed in CARES Act funding, with 24% of matched hospitals receiving less than $5 million in funding and 8% receiving more than $50 million. Academic-affiliated hospitals, hospitals with higher pre–COVID-19 assets and hospitals with higher COVID-19 cases received higher levels of funding, while critical access hospitals received lower levels of financial assistance. A 10% increase in hospital assets, endowment size, and COVID-19 cases was associated with 1.4% (95% CI, 0.8% to 2.0%; *P* = .003), 0.2% (95% CI, 0.1% to 0.3%; *P* < .001), and 3.5% (95% CI, 2.8% to 4.2%; *P* < .001) increases in CARES Act funding, respectively.

**Conclusions and Relevance:**

In this cross-sectional study of US hospitals and health systems, findings suggest that High-Impact Distribution CARES Act funds may have disproportionately gone to hospitals that were in a stronger financial situation prior to the pandemic compared with those that were not, but funds also went disproportionately to those that eventually had the most cases.

## Key Points

**Question:**

What is the association between financial assistance through the High-Impact Distribution Coronavirus Aid, Relief, and Economic Security (CARES) Act program and hospital-level financial resources?

**Findings:**

In this cross-sectional study among 952 hospital-level entities, wide ranges existed in CARES Act funding, with 24% of hospitals receiving less than $5 million in funding and 8% receiving more than $50 million. Academic-affiliated hospitals with higher pre–COVID-19 assets and hospitals that had higher COVID-19 cases received higher levels of funding, while critical access hospitals received lower levels of financial assistance.

**Meaning:**

CARES Act funds may have disproportionately gone to hospitals that were in a stronger financial situation prior to the pandemic compared with those that were not, but funds also went disproportionately to those that eventually had the most cases.

## Teaser

This cross-sectional study examines the disbursement of the High-Impact CARES Act funds and the association between financial assistance and hospital-level financial resources prior to the COVID-19 pandemic.

## Introduction

The COVID-19 pandemic has caused immense disruption to health care utilization and, in turn, hospital finances.^1^ Between February and April 2020, there was a decline of 20% for primary admissions, which slightly rebounded to 16% by early July 2020.^2^ The decline in volume could be due to multiple factors, including the suspension of elective and noncritical care in multiple states.^3^ Data from employer-sponsored claims data have found in the first 2 months of the COVID-19 pandemic dramatic reductions in the use of preventive and elective care.^4^ From April to June of 2020, estimates of hospital procedures were still on the decline for most procedure types.^5^ Recent survey data collected in June 2020 indicate that an estimated 41% of US adults delayed or avoided medical care owing to concerns about COVID-19.^6^

It has been reported anecdotally that because of the decline in hospital admissions, many hospitals may be forced to furlough employees or initiate salary cuts in an effort to reduce costs.^7^ Prior to the pandemic, labor and capital costs accounted for approximately 41% and 4% of hospital operating costs, respectively, and around a third of operating costs were from operating rooms, laboratories, diagnostic radiology, general routine inpatient care, intensive care units, outpatient clinics, and emergency departments.^8^ Early in the pandemic, data from the US Bureau of Labor Statistics showed that the number of hospital employees had decreased by 135 000 between March and April 2020, which constitutes a 2.6% decline.^9^

In response to decreased patient volume and weak financial positions, Congress passed the Coronavirus Aid, Relief, and Economic Security (CARES) Act, which provided funding for health care professionals that included an increase in payments by Medicare to eligible health professionals.^10^ The Provider Relief Fund provided $175 billion to reimburse eligible hospitals and health professionals for health care–related expenses or losses in revenue that were the result of COVID-19. As of May 31, 2020, 380 000 payments were made that totaled $65.2 billion.^10^ Of that $65.2 billion, the CARES Act budgeted $22 billion in targeted distributions for more than a thousand hospitals in high-impact areas that were most heavily affected by COVID-19 in the first surge of the pandemic.^11^ These funds were distributed automatically in 2 rounds under the COVID-19 High-Impact Distribution budget line item. As of October 21, 2020, more than 97% of budgeted funds had been disbursed in response to the first surge of COVID-19 cases. General distribution payments were dispersed according to a formula set by the Centers for Medicare & Medicaid Services, based on the hospital’s share of fee-for-service Medicare payments out of total Medicare payments in 2019 for the initial disbursement, and a function of the most recent annual gross receipts minus the initial disbursement.^11^ These funds were meant to support hospitals particularly hard hit financially by COVID-19 through well-documented decreases in volumes of care. However, it is not clear if historical measures of Medicare payments are the most appropriate indicator for hospital financial assistance need.^12^ In contrast, high-impact funds were disbursed based on hospital COVID-19 cases.^13^

While it is known what the funding allocation formulas are, it is unclear how these funds were targeted to hospitals in relation to their pre–COVID-19 finances, which is an important policy question to inform future resource allocations.^14^ Existing studies have found that initial funding allocations disproportionally went to hospitals with more cash on hand.^15^ This cross-sectional study examines the disbursement of CARES Act funding to hospitals and how funding allocations vary by hospital financial status and patient composition prior to the pandemic.

## Methods

### Hospital Cost Reports

Hospital-level data were from 2018 and were taken Hospital Cost Reports from the Healthcare Cost Report Information System (HCRIS).^16^ The HCRIS data contain detailed hospital information such as facility characteristics, utilization, costs, and charges for hospitals and cost centers.^17^ Financial data, including assets and investment income, were extracted and used to calculate prices and patient share by payers. Mean hospital prices were calculated as the revenue per discharge equivalent for Medicare (including traditional Medicare and Medicare Advantage), Medicaid, and commercial payers. Total hospital revenues include revenue from inpatient and outpatient settings. Discharge equivalents were calculated by the product of inpatient discharges and the ratio of total to inpatient hospital operating expenses.

### CARES Act Funding

CARES Act Provider Relief Fund payment records from 1193 hospitals through the High-Impact Distribution Funds program were analyzed.^13^ Nine hospitals were excluded that had already paid back all of their high-impact distribution funds. One double payment to a hospital that plans to pay back $62.6 million was also excluded.^18^

### COVID-19 Cases

Centers for Disease Control and Prevention data were used to calculate the total number of suspected COVID-19 hospitalizations through June 1, 2021.^19^ These hospitals were merged with the hospitals receiving high-impact funds to examine the association between funding and eventual COVID-19 case counts.

### Matching Hospital Cost Reports and CARES Act Fund Data

The HCRIS data and CARES Act Fund data were linked for a total of 1027 CARES Act payments matched to hospitals. The analysis included either individual hospitals, grouped hospitals, or grouped systems based on how funds were distributed. CARES funding data included the hospital’s name, city, and state, which were used to match to hospitals in the HCRIS database. Matching was done either by exact match by name-city-state (332 matches) or by manual matching and confirmation. When a hospital-level match occurred between the CARES data and HCRIS data, the hospital was the unit of analysis. Sometimes, 2 or more hospital names or organizations (such as a children’s hospital co-located with a general hospital) were merged into 1 unit of hospital-level analysis. Whenever none of the above matches were possible, we grouped hospitals by system to match with HCRIS hospital systems. The 157 hospitals that could not be matched were dropped from the analysis. Those excluded hospitals accounted for 8% of all CARES high-impact funding. This study was determined to not be human participants research by the RAND Institutional Review Board.

### Statistical Analysis

Linear regression models were used to assess the hospital characteristics associated with CARES Act funding. To reduce the influence of outliers and allow for a percentage comparison, the primary results used logarithmic-transformed CARES Act funding and hospital financial characteristics. Because log-transformed measures were used, the results can be interpreted in percentage changes by taking the exponent of the respective estimate. The regression models controlled for differences in patient composition using case-mix adjusted discharges for Medicare, Medicaid, and privately insured patients. Estimated regression models that test for differences based on the type of hospital (eg, teaching, nonprofit, and critical access) were also calculated. To account for differences in hospital size and patient population, all analyses were weighted by the number of discharges at each hospital. All *P* values were 2-tailed and used the .05 level to determine statistical significance. All analyses were performed using Stata, version 16 (StataCorp LLC). The study followed the Strengthening the Reporting of Observational Studies in Epidemiology (STROBE) reporting guideline.

## Results

A total of 952 hospital-level entities were used to analyze CARES Act funding distributions for high-impact funds. The matching accounted for 92% of all CARES Act funding disbursement as of October 22, 2020. Hospitals received a mean of approximately $33.6 million in total CARES Act high-impact funding, most of which was received during the first payment round, followed by a smaller round 2 payment (Table). The mean first-round payments was $22.1 million, compared with $11.5 million for second-round payments.

**Table.** Descriptive Characteristics of Matched Hospital Sample^a^

Characteristic	Mean (SD)
Discharge equivalents	24 895 (25 847)
Medicare	9594 (9673)
Commercial	10 076 (11 250)
Medicaid	4488 (5797)
**Price, $**	
Commercial	24 622 (18 906)
Medicare	15 286 (4994)
Medicaid	12 411 (7670)
Assets, $	
Total	696 000 000 (1 650 000 000)
Endowment	118 400 000 (60 220 000)
Other investment income	2 372 598 (15 700 000)
CARES payment, $	
1st	22 100 000 (209 000 000)
2nd	11 500 000 (53 400 000)
Total	33 600 000 (259 000 000)
**Discharge share, %**	
Commercial	0.405 (0.089)
Medicare	0.385 (0.080)
Medicaid	0.180 (0.103)
**Revenue share, %**	
Commercial	0.537 (0.126)
Medicare	0.334 (0.082)
Medicaid	0.129 (0.095)

Abbreviation: CARES, Coronavirus Aid, Relief, and Economic Security Act.

^a^ Sample is restricted to hospitals that received CARES Act Funds that were matched to Centers for Medicare & Medicaid Services Hospital Cost Report data.

**Figure 1.**
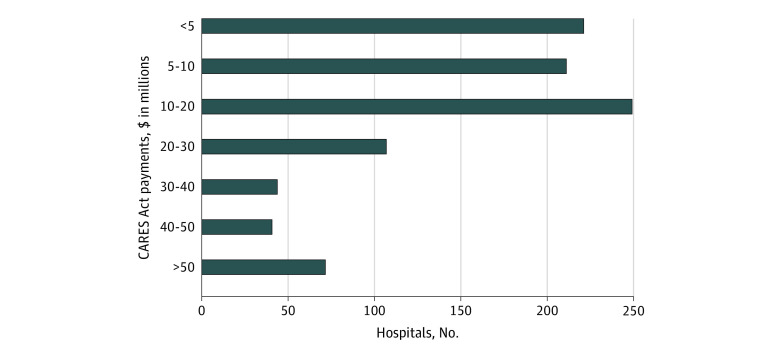
Distribution of CARES Act Funding Authors’ analysis of matched Coronavirus Aid, Relief, and Economic Security (CARES) Act data.

Figure 1 presents the distribution of CARES Act funding. Among the matched hospitals, 23.6% received less than $5 million in funding, 22.1% received between $5 and $10 million, 26.3% received $10 to $20 million, 11.4% received $20 to $30 million, 4.6% received $30 to $40 million, 4.3% received $40 to $50 million, and 7.8% received more than $50 million.

**Figure 2.**
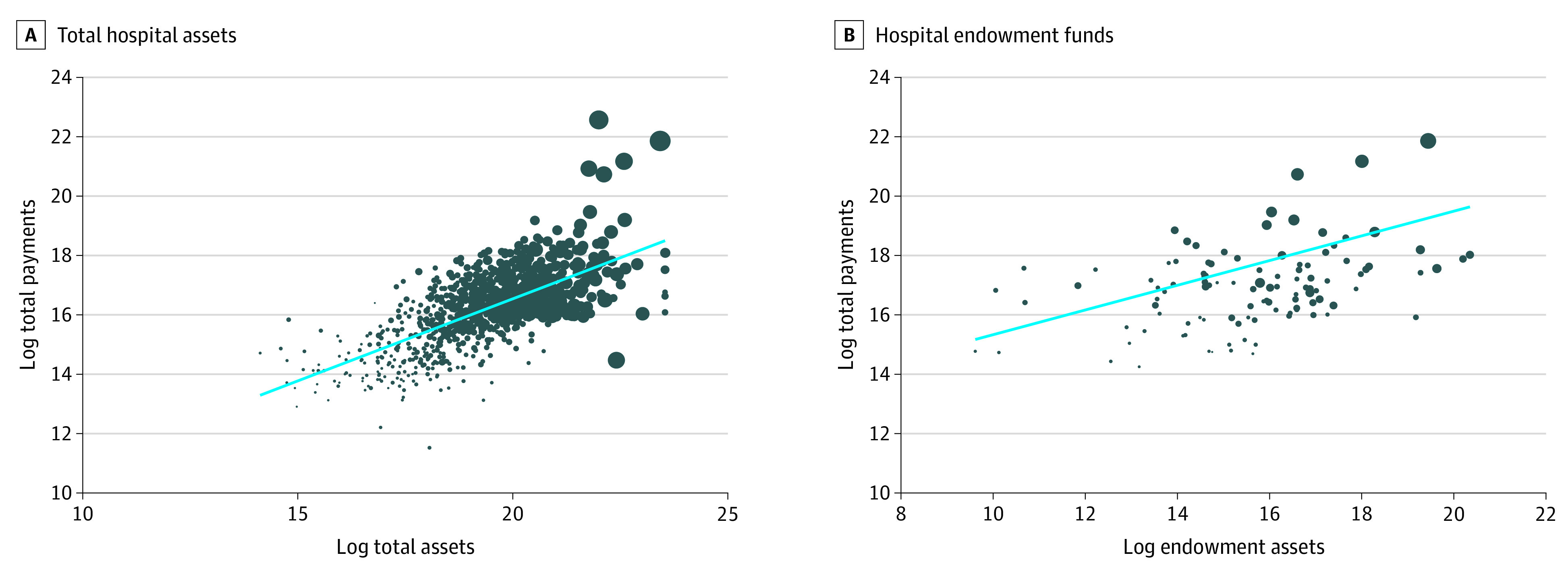
Distribution of Log-Transformed CARES Act Funding and Hospital Assets A, Total hospital assets; B, Hospital endowment funds. Authors’ analysis of matched Coronavirus Aid, Relief, and Economic Security (CARES) Act and Centers for Medicare & Medicaid Services Hospital Cost Report data. The dot weighting reflects total discharge equivalents for each hospital. The blue line is the regression line between the 2 variables. For A, the slope is 0.61; for B, 0.42.

Figure 2 presents the distribution of log-transformed CARES Act funding and hospital assets. Each hospital observation was weighted by the annual number of patient discharges. As reported in panel A, there was a high correlation between logged hospital assets and logged CARES Act payments. A 10% increase in hospital assets prior to the pandemic was associated with a 5.3% increase in CARES Act payments. Panel B presents similar results for hospital endowment funds. Among hospitals with endowment funds, a 10% increase in endowment funds was associated with a 2.6% increase in CARES Act funding.

**Figure 3.**
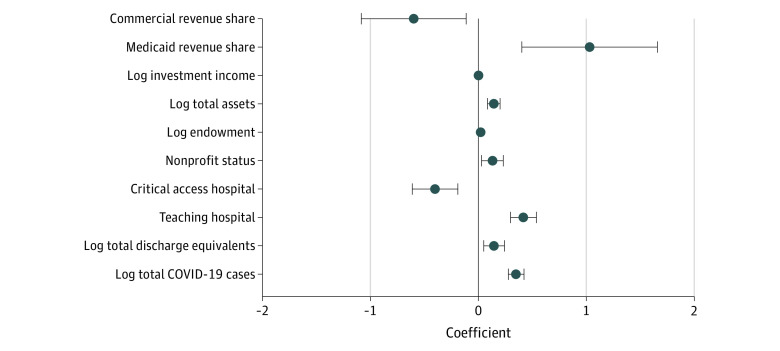
Association Between CARES Act Funding and Hospital Characteristics Analysis of matched Coronavirus Aid, Relief, and Economic Security (CARES) Act, Centers for Medicare & Medicaid Services Hospital Cost Report, and COVID-19 case data. Commercial revenue share (coefficient: −0.6; 95% CI, −1.1 to −0.1; *P* = .16); Medicaid revenue shares (1.0; 0.4 to 1.7; *P* < .001); log investment income (−0.0008; −0.0074 to 0.0058; *P* = .81); log total assets (0.1; 0.08 to 0.20; *P* < .001); log endowment (0.02; 0.01 to 0.03; *P* < .001); nonprofit status (0.13; 0.03 to 0.23; *P* = .01); critical access hospital (−0.40; −0.61 to −0.19; *P* < .001); teaching hospital (0.4; 0.3 to 0.5; *P* < .001); log total discharge equivalents (0.15; 0.05 to 0.24; *P* = .01); log total COVID-19 cases (0.35; 0.28 to 0.42; *P* < .001).

Figure 3 presents multivariate regression results that test the association between hospital characteristics and CARES Act funding. Hospitals with higher shares of total revenue coming from Medicaid revenues prior to the pandemic received a greater amount of CARES high-impact funding, while those with higher shares of total revenue coming from commercial revenues prior to the pandemic received a smaller amount of CARES high-impact funding. When accounting for differences in patient composition and the total number of hospital discharges, a 10% increase in hospital assets and endowment size prior to the pandemic and eventual COVID-19 case counts was associated with a 1.4% (95% CI, 0.8% to 2.0%; *P* = .003), 0.2% (95% CI, 0.1% to 0.3%; *P* < .001), and 3.5% (95% CI, 2.8% to 4.2%; *P* < .001) increases in CARES Act funding, respectively. Nonprofit hospitals received 13% (95% CI, 2.9% to 23%; *P* = .12) more CARES Act assistance, teaching hospitals received 42% (95% CI, 30% to 56%; *P* < .001) higher funding, and critical access hospitals received 40% (95% CI, −61% to −19%; *P* < .001) less CARES Act funding.

## Discussion

The COVID-19 pandemic created a massive shock to the US health care delivery system. In response to both concerns of infection transmission and local ordinances, many patients avoided receiving care. The lack of care translates to reduced revenue for many health care delivery organizations. In response to lower revenues, Centers for Medicare & Medicaid Services provided financial assistance to hospitals through the CARES Act.

Linked CARES Act funding data with hospital cost report data were used to document variations in CARES Act funding across hospitals. Regression analysis indicated that while more-resourced hospitals, measured through financial assets prior to the pandemic, did receive higher levels of CARES Act funding, the largest component of CARES Act funding distribution was the share of hospital revenue that occurs from Medicaid patients. Teaching hospitals also received higher levels of financial support, while critical access hospitals received lower levels of financial assistance. This disparity in funding may be of particular interest because many critical access and rural hospitals faced financial pressures even before the COVID-19 pandemic.^20^ Policy makers should continue to ensure that these types of hospitals are sufficiently funded, potentially with additional rounds of funding.

### Limitations

This study is not without limitations. First, while the measures of funding are comprehensive, they covered only the initial stage of the pandemic. It is expected that the costs to health care systems will be dependent on the length of the pandemic.^21^ Thus, updates to this analysis will be needed. Second, there may be systematic differences between hospitals that we were able to match between the HCRIS and CARES funds and those that we failed to match. For example, health care systems may have mechanically higher investments or assets and the HCRIS data pools data for an entire health care system while the CARES funds do not. Eight percent of CARES funds was unmatched in the sample. These failed matches may be systematically different, have different patient populations, and operate different Medicare systems. Finally, our analysis focused only on the High-Impact Distribution Funds program and did not assess other CARES Act funding provided to rural facilities ($11.1 billion) and safety-net hospitals ($13.1 billion), which may have benefited hospitals with fewer financial resources.^22^

Addressing the shock of the COVID-19 pandemic required a rapid response. Using a more nuanced approach than Medicare fee-for-service, such as based on total hospital assets or operating margins, may have differently allocated resources but would have likely delayed funding because Medicare Hospital Cost Reports are available at a 2-year delay. As the COVID-19 pandemic evolves, future work should examine the outcomes of differential CARES Act funding on hospital investments, technologies, and behavior.

## Conclusions

In this cross-sectional study of US hospitals assessing the characteristics of hospitals that received High-Impact Distribution CARES Act funding and the magnitude of CARES Act funding, wide variation existed in the funding distributed to hospitals. Academic hospitals and hospitals with higher pre–COVID-19 assets received higher levels of funding, while critical access hospitals received lower levels of financial assistance. The results suggest that CARES Act funds may have disproportionately gone to hospitals that were in a stronger financial situation prior to the pandemic compared with those that were not.
