# Errors in Introduction and Discussion

**DOI:** 10.1001/jamahealthforum.2021.4096

**Published:** 2021-11-19

The Original Investigation titled, “Association Between COVID-19 Relief Funds and Hospital Characteristics in the US,”^1^ published online October 22, 2021, included errors in the Introduction and Discussion sections. The text has been corrected to clarify the basis for general and high-impact funds distribution. This article has been corrected online.
